# Inhibition of hepatitis C virus by an M1GS ribozyme derived from the catalytic RNA subunit of Escherichia coli RNase P

**DOI:** 10.1186/1743-422X-11-86

**Published:** 2014-05-13

**Authors:** Xinliang Mao, Xifang Li, Xinjun Mao, Zhiwen Huang, Chengcheng Zhang, Wenjun Zhang, Jianguo Wu, Gang Li

**Affiliations:** 1 Vaccine Institute, The Third Affiliated Hospital of Sun Yat-sen University, Guangzhou 510630, PR China; 2 Department of Microbiology and Immunology, Guangdong Pharmaceutical University, Guangzhou Higher Education Mega Center, Guangzhou 510006, PR China; 3 Department of Anaesthesia, General Intensive Care and Pain Management, Medical University of Vienna, Vienna A-1090, Austria; 4 State Key Laboratory of Virology, Wuhan University, Wuhan 430072, PR China

**Keywords:** Ribozyme, RNase P, Hepatitis C virus, 5′ UTR, Antiviral

## Abstract

**Background:**

Hepatitis C virus (HCV) is a human pathogen causing chronic liver disease in about 200 million people worldwide. However, HCV resistance to interferon treatment is one of the important clinical implications, suggesting the necessity to seek new therapies. It has already been shown that some forms of the catalytic RNA moiety from *E. coli* RNase P, M1 RNA, can be introduced into the cytoplasm of mammalian cells for the purpose of carrying out targeted cleavage of mRNA molecules. Our study is to use an engineering M1 RNA (i.e. M1GS) for inhibiting HCV replication and demonstrates the utility of this ribozyme for antiviral applications.

**Results:**

By analyzing the sequence and structure of the 5′ untranslated region of HCV RNA, a putative cleavage site (C^67^-G^68^) was selected for ribozyme designing. Based on the flanking sequence of this site, a targeting M1GS ribozyme (M1GS-HCV/C_67_) was constructed by linking a custom guide sequence (GS) to the 3′ termini of catalytic RNA subunit (M1 RNA) of RNase P from *Escherichia coli* through an 88 nt-long bridge sequence. *In vitro* cleavage assays confirmed that the engineered M1GS ribozyme cleaved the targeted RNA specifically. Moreover, ~85% reduction in the expression levels of HCV proteins and >1000-fold reduction in viral growth were observed in supernatant of cultured cells that transfected the functional ribozyme. In contrast, the HCV core expression and viral growth were not significantly affected by a “disabled” ribozyme (i.e. M1GS-HCV/C_67_*). Moreover, cholesterol-conjugated M1GS ribozyme (i.e. Chol-M1GS-HCV/C_67_) showed almost the same bioactivities with M1GS-HCV/C_67_, demonstrating the potential to improve *in vivo* pharmacokinetic properties of M1GS-based RNA therapeutics.

**Conclusion:**

Our results provide direct evidence that the M1GS ribozyme can function as an antiviral agent and effectively inhibit gene expression and multiplication of HCV.

## Background

Hepatitis C virus (HCV), a member of the *Flaviviridae* family, causes chronic liver disease in about 200 million people worldwide. Its single-stranded RNA genome comprises a 5′ untranslated region (UTR), a long open reading frame and a 3′-noncoding region, and functions as the only mRNA species for translation. The 5′ UTR serves as an internal ribosome entry site (IRES), while the open reading frame encodes a polyprotein precursor (~3010 amino acids), which is cleaved into structural and nonstructural proteins [1]. HCV is known to cause persistent infection and result in severe liver damages, including cirrhosis, hepatic steatosis and hepatocellular carcinoma [2]. Current clinical approaches are unable to provide satisfactory therapy for HCV-infected patients. For example, the combination of interferon-alpha with ribavirin and/or viral protease inhibitors is effective only in 40% of infected population [3]. Moreover, the efficacy of treatment usually depends on the particular HCV genotype [4]. Therefore, novel antiviral agents and therapies are urgently needed.

**Figure 1:**
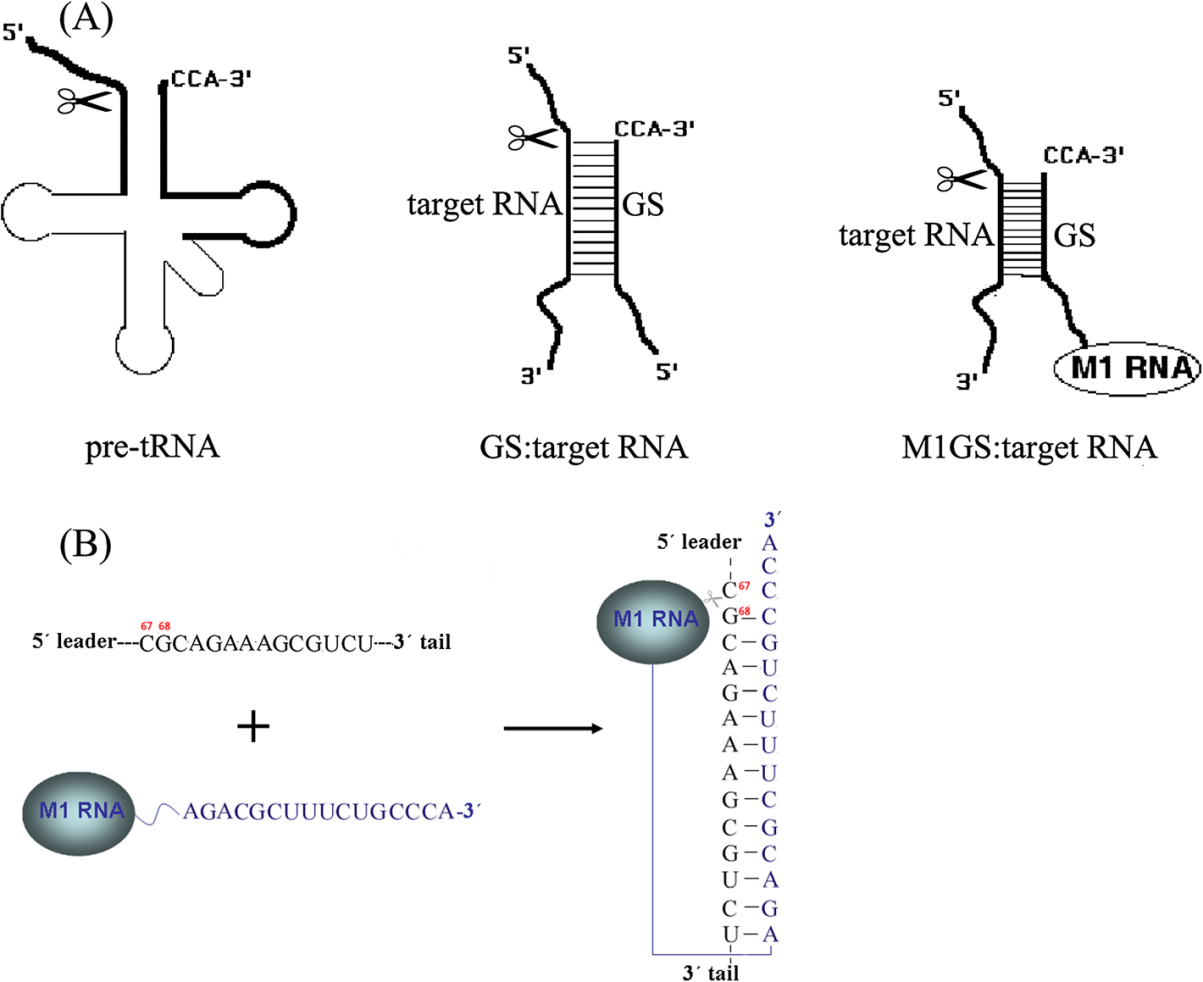
**Schematic representation of RNase P substrates. (A)** A natural substrate (pre-tRNA), a small model substrate (GS:target RNA) for M1 RNA from *E. coli*, and a complex structure formed between a M1GS RNA and its RNA substrate. The structural components common to pre-tRNA are highlighted. The site of cleavage by RNase P or M1 RNA is marked with scissors. **(B)** The substrate and M1GS ribozyme constructed in this study. The targeted sequence and guide sequence of the ribozyme are lettered in black and in blue, respectively.

Among the proposed antiviral agents are the ribozyme derived from ribonuclease P (RNase P), which is a ribonucleoprotein complex with ribozyme activity that catalyzes a hydrolysis reaction to remove the leader sequence of pre-tRNA and generate mature tRNA [5]. In *Escherichia coli*, RNase P consists of an RNA subunit (M1 RNA) and a protein subunit (C5 protein). The catalytic M1 RNA can cleave the pre-tRNA substrate *in vitro* at high divalent ion concentrations in the absence of C5 protein [6]. Moreover, M1 RNA is able to cleave a target RNA sequence efficiently if an additional small RNA is covalently linked to the 3′ end of M1 RNA. The new sequence-specific ribozyme is named M1GS ribozyme (Figure 1A) [7,8], and any RNA could in principle be targeted by a custom-designed M1GS for specific cleavage. When introduced into human cells, M1GS ribozyme can function independently from the endogenous human RNase P to cleave a targeting sequence that base pairs with the guide sequence [9]. A number of studies have shown that M1GS RNA and RNase P are effective to cleave both viral and cellular mRNAs and block their expression in cultured cells, including inhibition of gene expression of human influenza, herpes viruses, human cytomegalovirus and human immunodeficiency virus [10-15]. Therefore, M1GS-based strategy is not only a useful method in basic research (e.g. regulation of gene expression) but also represents a distinctive therapeutic approach of nucleic-acid-based, sequence-specific interference [16].

By using RNase P as a tool, the genomic RNA of HCV and the related animal pestiviruses has been previously found to be directly processed *in vitro* by wild type RNase P of either human origin or *Synechocistis* sp but not by the wild type *E. coli* RNase P ribozyme (M1 RNA) [17-20]. Therefore, we attempt to use the M1GS ribozyme to develop a new anti-HCV strategy and nucleic acid agent. In this study, we chose the 5′ UTR of HCV genome as the target region as it is relatively conserved and important for the initiation of viral polyprotein translation [21]. We show here that M1GS ribozyme was able to efficiently cleave the target RNA sequence *in vitro*. Also about 85% reduction of HCV core protein expression and ≥1000-fold reduction of viral growth were observed in cells transfected with the functional M1GS ribozyme.

## Materials and methods

### Viruses, cells and antibodies

HCV strain JFH1 (GT2a) was kindly provided by Dr. Takaji Wakita (National Institute of Infectious Disease, Tokyo, Japan). Huh7.5.1 cells were kindly provided by Dr. Francis Chisari (The Scripps Research Institute, California, USA) and were cultured in Dulbecco’s modified Eagle’s medium (DMEM) supplemented with 10% fetal calf serum (FCS), 100 U/ml penicillin and 100 μl/ml streptomycin sulfate. The monoclonal antibody MBS140205, which reacts with HCV core protein, was purchased from MyBioSource, Inc (San Diego, USA). The monoclonal antibody against human GAPDH was purchased from Guangzhou Whiga Technology Co., Ltd (Guangzhou, China).

### Ribozyme and substrate constructs

By analyzing the sequence and structure of the 5′ UTR of HCV RNA, a putative cleavage site (C^67^-G^68^) was selected for designing ribozyme. Based on the flanking sequence of this site, a targeting M1GS ribozyme (M1GS-HCV/C_67_) was constructed by linking a custom guide sequence (GS) to the 3′ termini of catalytic RNA. Plasmid pFL117, which contains the DNA sequence encoding M1 RNA driven by the T7 RNA polymerase promoter, was a gift from Prof. Fenyong Liu (University of California, Berkeley). The DNA sequences that encode ribozymes M1GS-HCV/C_67_ and M1GS-HCV/C_67_* were constructed by PCR using the DNA sequence of M1 RNA as templates, with oligonucleotides P1 (5′- CGGAATTCGAAGCTGACCAGACAG-3′) as the forward primers, and P2 (5′- CCCAAGCTTGGTGTATAGCCATGGCTGTGGAATTGTGAGCG-3′) and P2*(5′- CCCAAGCTTGGTGTATAGCCATGGCAGGTGAAACTGACCGA-3′) as the reverse primers, respectively. The two PCR products were further inserted into the multiple cloning site of the vector (pUC19) between the restriction sites *Eco*R I and *Hin*d III, and two recombinant plasmids containing gene of M1GS ribozyme were constructed, i.e. pM1GS-HCV/C_67_ and pM1GS-HCV/C_67_*, respectively. The DNA sequence that encodes substrate S_1–584_ was constructed by PCR using plasmid pGEM-HCJ4 (a gift from Prof. Zhongtian Qi, The Second Military Medical University, China) as template, and oligonucleotides P3 (5′-CGGAATTCGCCAGCCCCCTGATGG-3′) and P4 (5′-CGGGATCCGACGCCATCGCCGATGCGGGGCGATCCTATAGTGAG-3′) as forward and reverse primers, respectively. The PCR product was then inserted into the multiple cloning site of vector pGEM3z (between restriction sites *Eco*R I and *Hin*d III), and a recombinant plasmid containing DNA sequence that encodes the substrate (S_1–584_) was constructed, i.e. pGEM-S. Another irrelevant substrate, segment of HCMV UL97 mRNA (1-600 nt), was selected as a control of cleavage assay. The corresponding recombinant plasmid was subcloned in a previous study [22].

### In vitro cleavage by M1GS RNA

The M1GS RNAs and S_1–584_ RNA substrate were synthesized *in vitro* with T7 RNA polymerase (Takara Biotechnology Co., Ltd, Dalian, China) according to the manufacture’s recommendations and purified on 8% urea/polyacrylamide gels. In addition, a cholesterol-modified M1GS RNA (i.e. Chol-M1GS), which conjugated a cholesterol molecule to the 5′ terminus of M1 RNA through a pyrrolidine linker, was synthesized (Guangzhou RiboBio Co., Ltd., China). Subsequently, the M1GS RNAs (10 nM) were mixed with the [^32^P]-labeled RNA substrate (10 nM). The cleavage reactions were carried out at 37°C in a volume of 10 ul for 30 min in a buffer consisting of 50 mM Tris (pH 7.5), 100 mM NH_4_Cl and 100 mM MgCl_2_. Reaction was stopped by the addition of 10 ul solution containing 7 mol/L urea, 0.05% bromophenol blue and 0.05% xylene cyanol. Cleavage products were separated in denaturing gels and quantitated with a Typhoon 9200 phosphorImager (Amersham Biosciences).

### M1GS RNA internalization and viral infection

Huh7.5.1 cells were seeded at a density of 5.0 × 10^5^ cells per well in 6-well plates and grown to approximately 80% confluence prior to transfection. M1GS RNAs were transfected into cells by using Lipofectamine 2000 reagent (Invitrogen). Lipofectamine 2000 reagent was diluted in 100 μl of Opti-MEM medium with the M1GS to give a final concentration of 10 μg/ml lipid-100 nM M1GS RNA. The transfection experiments were carried out by using 100 nM M1GS RNA. At 12 h post-transfection, cells were serum starved for 12 h and then infected (or mock-infected) by HCV (strain JFH1) at a multiplicity of infection (MOI) of 1–5 in an inoculum of 1.5 ml DMEM supplemented with 1% FCS. After 2 h of exposure to the virus at 37°C, the inoculum was replaced with DMEM supplemented with 10% FCS.

### Inhibition assay of viral gene expression by M1GS ribozyme

To measure the inhibition of viral gene expression by M1GS ribozyme, viral RNA and core protein were detected by Northern blotting and Western blotting, respectively. Cells were harvested at 48 h post-infection. For Northern blotting, RNA extract was prepared as described previously [22]. The RNA fractions were separated in 1% agarose gels containing formaldehyde, transferred to a nitrocellulose membrane, hybridized with ^32^P-labeled DNA probes that contained the cDNA sequences of HCV core coding region, M1 RNA gene or human β-actin gene, and analyzed with a Typhoon 9200 PhosphorImager. The radiolabeled DNA probes were used to detect HCV RNA (9.6 kb), M1GS RNAs (~0.48 kb) and human β-actin mRNA, respectively. For Western blotting, the cells were harvested and washed twice with phosphate-buffered saline (PBS), and lysed in disruption buffer consisting of 0.05 M Tris (pH 7.0), 8.5% (w/w) sucrose, 5% (w/w) β-mercaptoethanol and 2% (w/w) sodium dodecyl sulfate. The protein samples were boiled for 5 min before electrophoretic separation on 9% (w/w) SDS-polyacrylamide denaturing gels cross-linked with N,N’-methylenebisacylamide. The separated polypeptides were transferred electrically to PVDF membranes and reacted to the antibodies against HCV core protein and human GAPDH. The membranes were subsequently stained with a chemiluminescent substrate using a Western chemiluminescent substrate kit (Bestbio Company, Shanghai) and quantitated with a Typhoon 9200 phosphorImager. Quantitation was performed in the linear range of protein detection.

### Inhibition assays of viral growth by M1GS ribozyme

Huh7.5.1 cells (n =5 × 10^5^) were infected with HCV at an MOI of 1, and harvested at 4, 24, 36, 72 and 96 h post-infection. The HCV RNA copies in the medium were respectively determined by fluorescence quantitative PCR, which was performed in a LightCycler 480 thermal cycler (Roche) under the following conditions: heat activation of the polymerase for 5 min at 95°C, followed by 40 cycles of 95°C for 15 sec, 55°C for 15 sec and 72°C for 20 sec. The final melting curve was measured from 50°C to 95°C. The primers for HCV RNA were 5′-CGTTCTTGCGTCCTTCATCT-3′ (forward) and 5′-CACAAAGTAGGGCTTGGTCAT-3′ (reverse). The primers for β-actin (internal standard) were 5′-TCGTCCACCGCAAATGCTTCTAG-3′ (forward) and 5′-ACTGCTGTCACCTTCACCGTTCC-3′ (reverse).

## Results

### Construction of M1GS ribozymes

**Figure 2:**
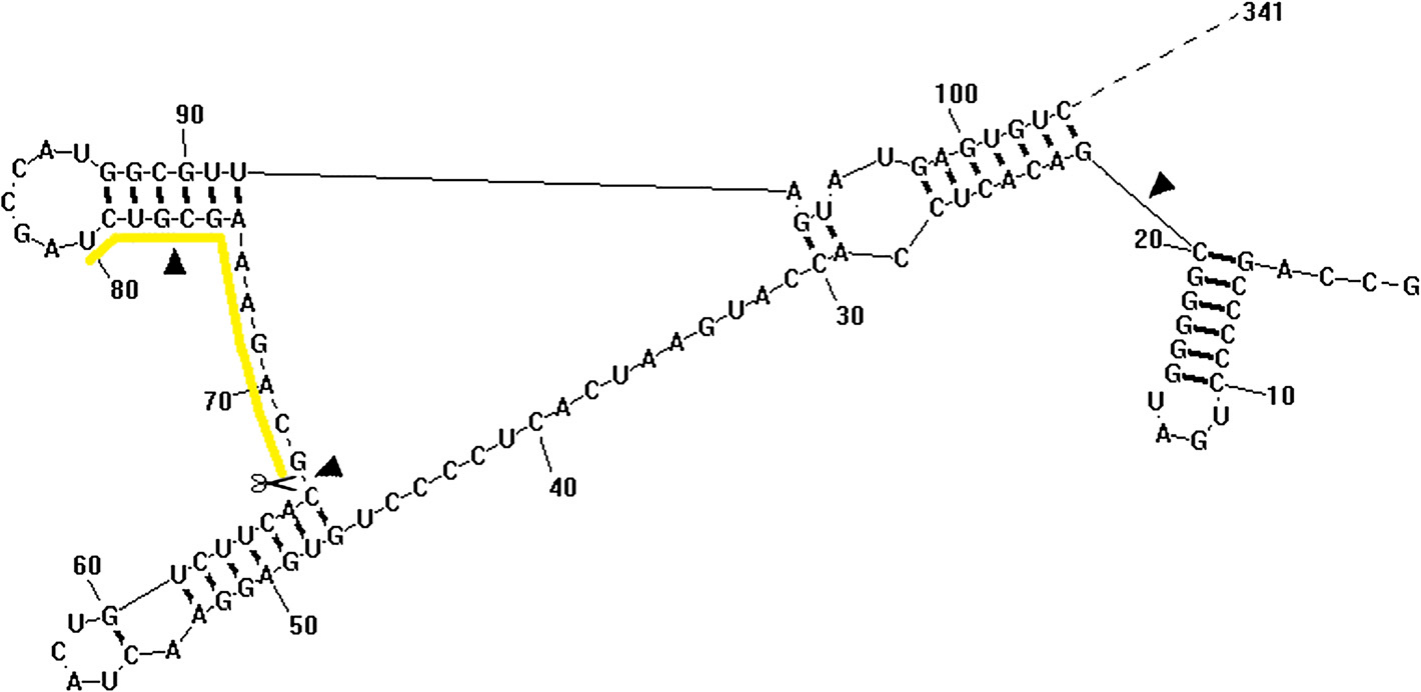
**Secondary structure of a portion of HCV 5′ UTR predicted by the RNA Structure software.** The three sites indicated by triangular arrows were potential cleavage sites of M1GS ribozyme. The site indicated by scissors was an optimal cleavage site, with the sequence marked by a yellow line capable of forming a long single-strand structure and facilitating complementary binding with the GS sequence of M1GS ribozyme.

The 5′ UTR of HCV genomic RNA consists of 341 nucleotides and folds to six secondary structure domains termed SLI-VI. SLII, III and IV form an IRES that facilitates the translation of capless HCV RNA [23]. IRES is present in a highly organized conformation and associated with proteins inside cells. In order to achieve efficient targeting, it is pivotal to choose a targeting region which is accessible to binding of ribozymes. Therefore, DNAMAN (v. 6) and RNA structure (v. 4.5) softwares were used to analyze the sequence and secondary structure of HCV 5′ UTR to search for sequences with recognition and cleavage features of M1GS ribozyme as characterized previously, including requirement for a guanosine and a pyrimidine to be the 3′ and 5′ nucleotides adjacent to the site of cleavage, respectively [24]. It was found that three sites were critical for the cleavage by M1GS ribozyme, and a position 67 nucleotides downstream from the first nucleotide of HCV genome was chosen as the cleavage site for M1GS RNA because this site appears to be one of the regions most accessible to HCV RNA in the 5′ UTR. Moreover, the flanking sequence of the site also exhibited several features needed to interact with an M1GS ribozyme to achieve efficient cleavage (Figure 2).

**Figure 3:**
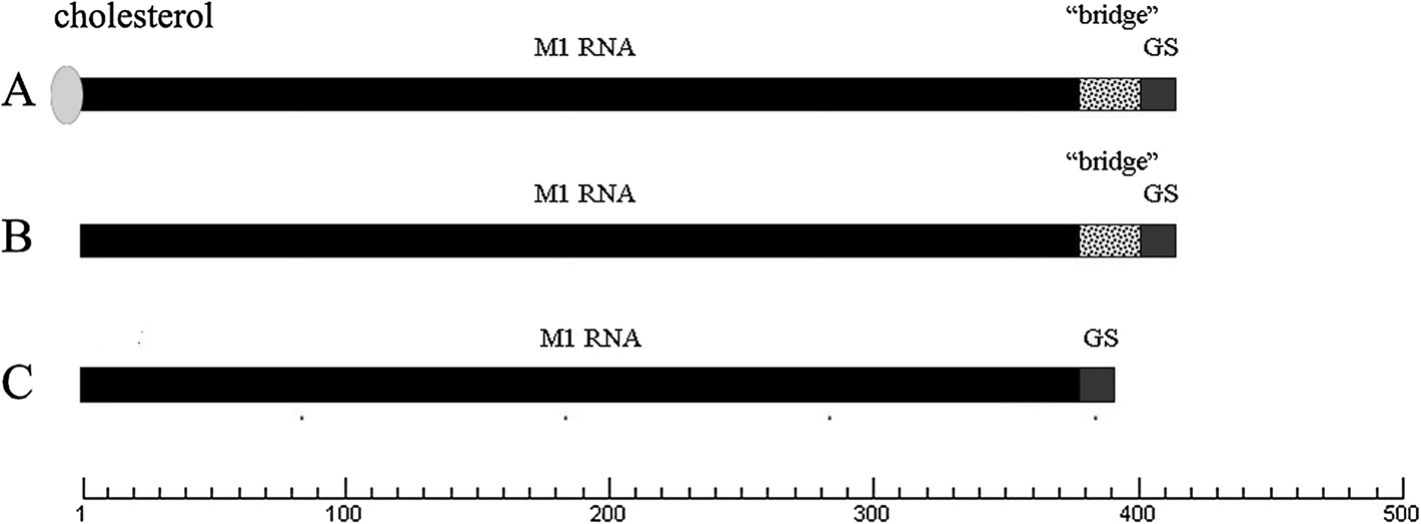
**Schematic structure of the DNA sequences encoding M1GS ribozymes used in this study. (A)** M1GS ribozyme with its guide sequence (GS) directly linked to the 3′ terminus of M1 RNA. **(B)** M1GS ribozyme containing a bridge sequence, which is of 88 nt-length and links a GS to the 3′ terminus of M1 RNA. **(C)** Cholesterol-modified M1GS ribozyme containing a cholesterol molecule to the 5′ terminus of M1 RNA, with a bridge sequence between GS and M1 RNA.

**Figure 4:**
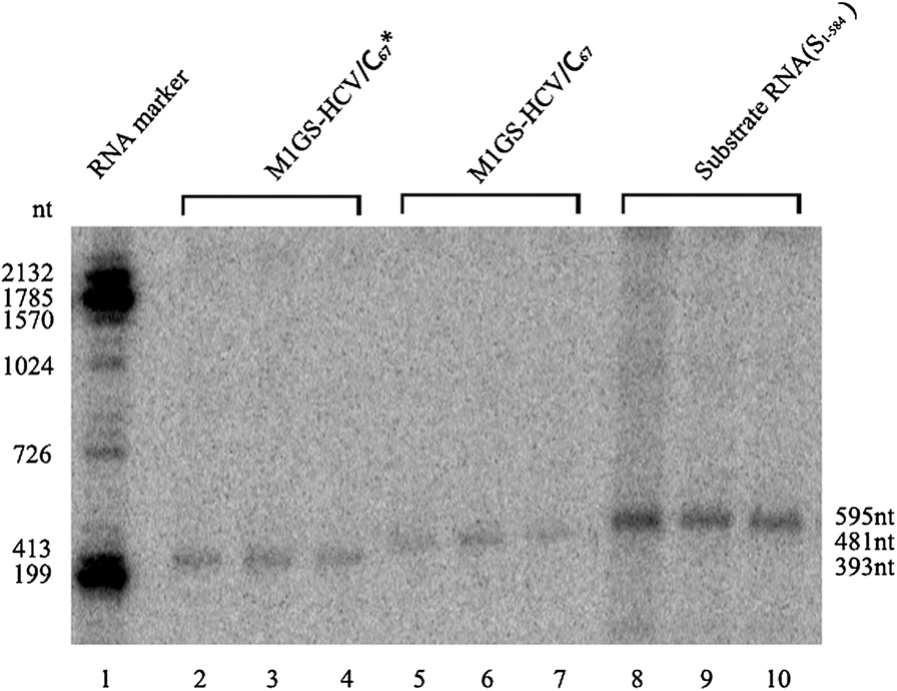
**Determination of M1GS ribozyme transcribed *in vitro *by T7 RNA polymerase.** The ^32^P-labeled M1GS RNAs and ^32^P-labeled substrate RNA (S_1–584_) transcribed *in vitro* were separated on 15% polyacrylamide gels containing 8 M urea.

According to the flanking sequence of the chosen site, two M1GS ribozymes, i.e. M1GS-HCV/C_67_ and M1GS-HCV/C_67_*, were constructed by covalently linking the 3′ termini of M1 RNA with a 13-nucleotide GS that was complementary to the targeted RNA sequence. As shown in Figure 3, M1GS-HCV/C_67_ contained a bridge sequence between GS and M1 RNA, whereas M1GS-HCV/C_67_* ribozyme had no bridge sequence – the same GS was directly linked to the 3′ termini of M1 RNA without a linker. Then the DNA fragments containing M1GS genes were inserted in *Eco*R I/*Hin*d III sites of pUC19, and the two recombinant plasmids pM1GS-HCV/C_67_ and pM1GS-HCV/C_67_* thus obtained were determined by PCR and double endonuclease digestion, and further verified by sequencing (data no shown). To obtain M1GS RNAs, pM1GS-HCV/C_67_ and pM1GS-HCV/C_67_* were first *Hin*d III-linearized and then synthesized *in vitro* by T7 RNA polymerase. The transcripts of M1GS-HCV/C_67_ and M1GS-HCV/C_67_* ribozymes were confirmed to be 481 nt and 393 nt, respectively (Figure 4).

### *In vitro* cleavage activity of M1GS ribozymes

**Figure 5:**
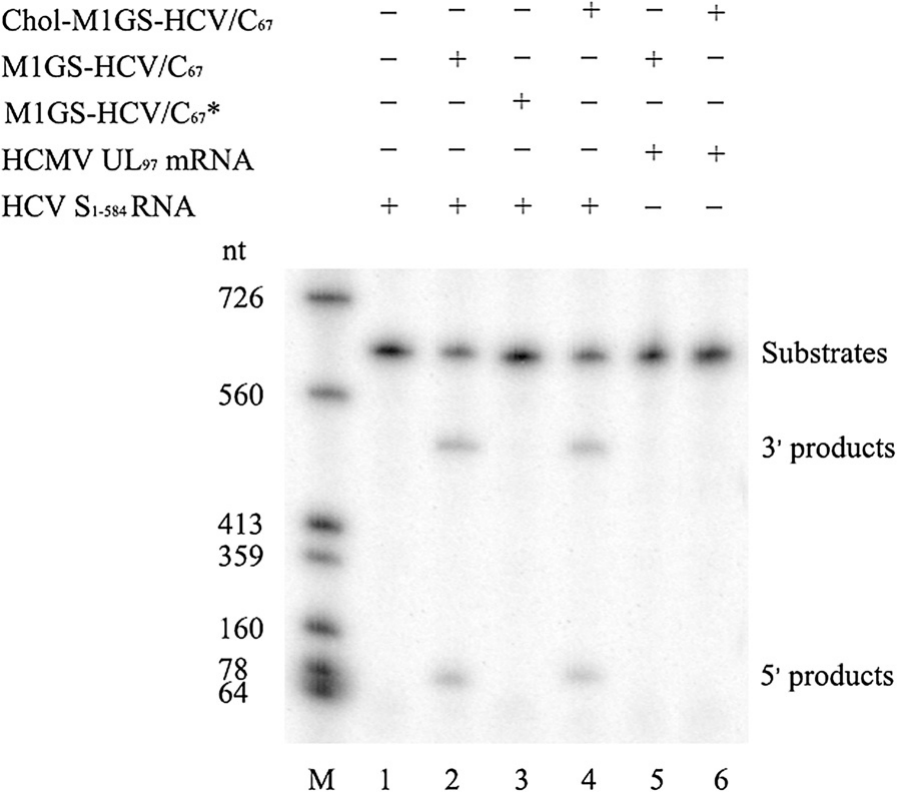
***In vitro *cleavage of the targeted RNA by M1GS ribozymes.** No ribozyme was added to the reaction mixture in lane 1; 10 nM of M1GS-HCV/C_67_ (lanes 2), M1GS-HCV/C_67_* (lanes 3) and Chol-M1GS/C_67_ (lane 4) were incubated with ^32^P-labeled substrate RNA (S_1–584_, 10 nM) at 37°C in a volume of 10 μl for 30 min in cleavage buffer. As a control, equal volumes of M1GS-HCV/C_67_ and Chol-M1GS-HCV/C_67_ were separately incubated with another ^32^P-labeled substrate RNA (HCMV UL97 RNA) in the same condition (lanes 5 and 6) Cleavage products were separated on 15% polyacrylamide gels containing 8 M urea. The RNA makers were transcribed *in vitro* by T7 RNA polymerase with DNA templates linearized by different restriction enzymes (lane M).

S_1–584_, a substrate containing the front 584 nucleotides of HCV genomic RNA, was used to test the cleavage activity of M1GS ribozymes (Figure 4 Lanes 8–10). The ribozymes and RNA substrate were synthesized *in vitro* and then incubated for ribozyme cleavage assays. No cleavage of S_1–584_ was detected in the absence of M1GS RNA (Figure 5 Lane 1), while efficient cleavage of the substrate by M1GS-HCV/C_67_ or Chol-M1GS-HCV/C_67_ ribozyme yielding products of 67 and 528 nucleotides was observed (Figure 5 Lane 2 and 4). These results demonstrate that M1GS-HCV/C_67_ was a functional ribozyme and that cholesterol modification did not affect the *in vitro* cleavage activity of the ribozyme. However, cleavage of the same substrate by M1GS-HCV/C_67_* ribozyme was barely detected (Figure 5 Lane 3), suggesting M1GS-HCV/C_67_*, which contains the same antisense GS as M1GS-HCV/C_67_ but is catalytically inactive, can be used as a control for antisense effect in cultured cells.

### Inhibition of HCV gene expression in M1GS-transfected cells

**Figure 6:**
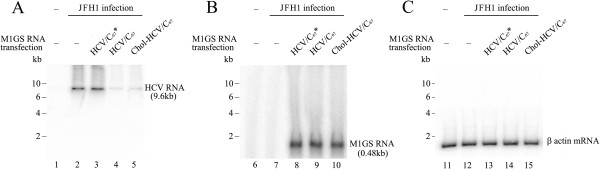
**Levels of HCV RNA as determined by Northern analysis.** Cells (n = 1 × 10^6^) were either mock-infected (lanes 1, 6 and 11) or infected with JFH1 (MOI = 5) and harvested at 48 h after infection. Northern analysis was carried out with RNA isolated from Huh7.5.1 cells transfected with M1GS-HCV/C_67_* (lanes 3, 8 and 13), M1GS-HCV/C_67_ (lanes 4, 9 and 14), and Chol-M1GS-HCV/C_67_ (lanes 5, 10 and 15). Equal amount of each RNA sample was separated on agarose gels containing formaldehyde, transferred to a nitrocellulose membrane, and hybridized to a ^32^P-radiolabeled probe containing the cDNA sequences of HCV core coding region **(A)**, M1 RNA gene **(B)**, and human β-actin gene **(C)**, respectively.

**Figure 7:**
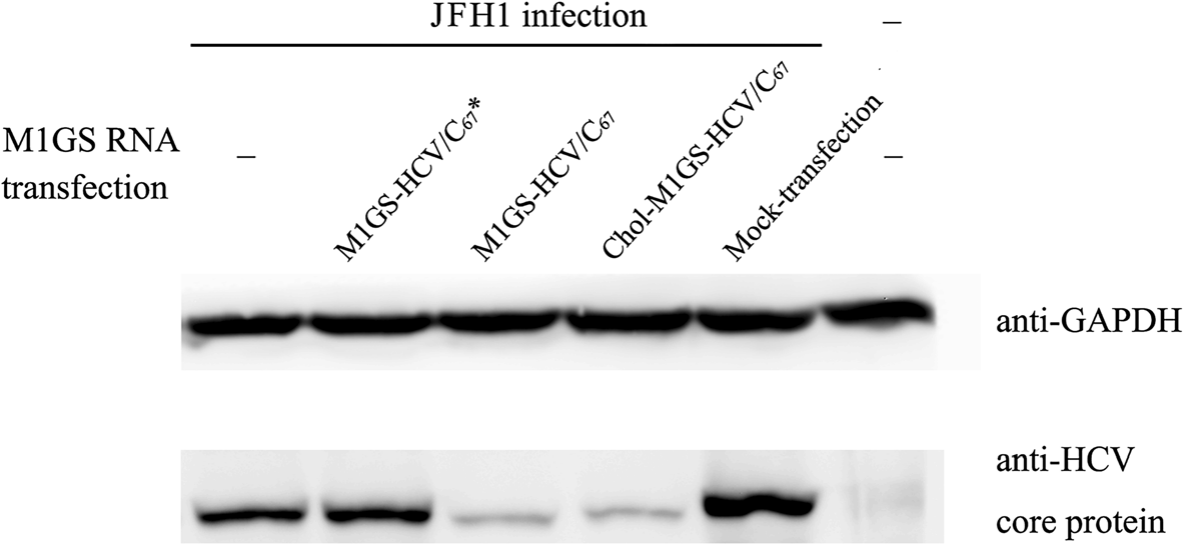
**Levels of HCV core protein as determined by Western blot analysis.** Cells were transfected with ribozymes or mock-transfected and further infected with JFH1 (MOI = 5; lanes 1–5) for 48 h. Protein samples were separated on 9% polyacrylamide gels, transferred to membranes, and analyzed with anti-HCV core protein and human GAPDH monoclonal antibodies.

To determine whether M1GS ribozymes inhibit HCV gene expression, Huh7.5.1 cells were first transfected with M1GS RNAs and then infected with JFH1 at a MOI of 5. Total cellular RNAs and proteins were prepared from the infected cells at 48 h post-infection. The levels of HCV RNA were determined by Northern analysis, with the M1GS RNA and β actin mRNA as internal controls (Figure 6B and C). Obvious reduction of the HCV RNA levels was observed in cells transfected with M1GS-HCV/C_67_ or cholesterol-conjugated M1GS-HCV/C_67_ (Figure 6A, Lanes 4 and 5), while almost no reduction was found in cells transfected with M1GS-HCV/C_67_* (Figure 6A, Lane 3). Furthermore, more than 85% reduction of HCV core protein level in cells transfected with M1GS-HCV/C_67_ or Chol-M1GS-HCV/C_67_ was also revealed by Western blotting with GAPDH as an internal control (Figure 7, Lane 3 and 4), while no obvious reduction was found in cells transfected with M1GS-HCV/C_67_* (Figure 7, Lane 5).

### Inhibition of viral growth by M1GS ribozyme

**Figure 8:**
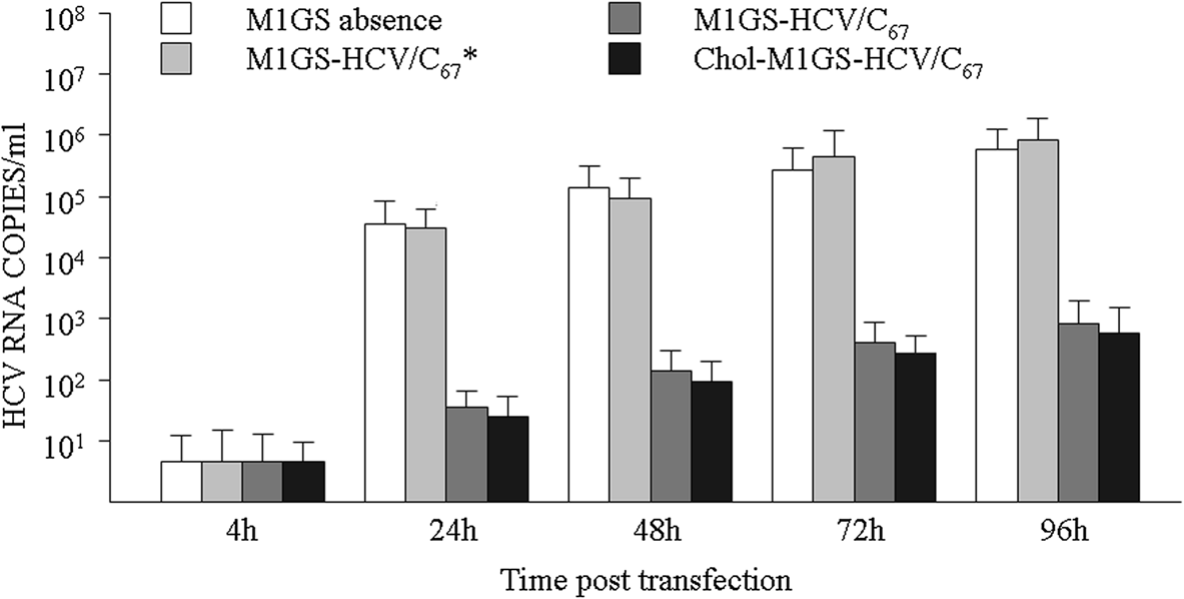
**Viral titers in supernatant of cultured cells that transfected with M1GS ribozyme.** Huh7.5.1 cells were infected with JFH1 at a MOI of 1. The culture supernatants were then harvested at the indicated times. Quantitation of HCV titer was determined from viral RNA copies by real-time PCR method. Data shown were the means from triplicate experiments.

To test whether HCV growth is inhibited by ribozyme transfection, cells were infected by JFH1 at an MOI of 1 and harvested at 4, 24, 36, 72 and 96 h post-infection. Viral RNA copies in the culture medium at the indicated times were determined. As shown in Figure 8, no significant reduction was found in the cell cultures transfected with M1GS-HCV/C_67_*, but >1000-fold reduction in viral yield was observed in supernatant of cultured cells transfected with M1GS-HCV/C_67_ or Chol-M1GS-HCV/C_67_ after 24 h of infection. These results suggest that M1GS-mediated targeting of viral RNA effectively inhibits HCV growth and cholesterol modification on M1GS ribozyme does not reduce its antiviral activity.

## Discussion

RNase P is an essential ribonucleoprotein complex found in all three domains of life. The RNase P holoenzyme is composed of an RNA subunit and one or more protein subunits. The RNA component is the catalytic moiety of RNase P across all phylogenetic domains, and is responsible for the maturation of 5′ termini of all pre-tRNAs, which account for about 2% of total cellular RNA [25]. A unique feature of RNase P is its recognition of substrate structures and thus is able to hydrolyze different natural substrates [26]. This is of great advantage because recognition of structures rather than sequences may help the fight against variable viruses as single or even double mutations in the target sequence may well be tolerated [27]. It is well known that HCV is highly variable, and it has been proved that HCV genome cannot be defined by a single sequence but by a population of closely related variant sequences [28]. In consideration of the “quasispecies” nature of HCV genome [29], the RNase P-based M1GS ribozyme is a promising antisense technique in HCV therapeutic studies.

HCV 5′ UTR is the most conserved locus within its genome and thus efforts related to HCV RNA therapeutics have been focused on this locus [30]. Nevertheless, this region has a highly stable RNA structure and is modulated by miRNAs and RNA-binding proteins, which limits the number of accessible sites for ribozyme targeting [31]. Therefore, it is important to select the target regions from the 5′ UTR of HCV RNA that are accessible to M1GS binding. In this study, we first analyzed the sequence of HCV 5′ UTR and found that three sites, i.e. C^20^-G^21^, C^67^-G^68^ and U^76^-G^77^, met the general features for M1GS cleavage activities [32]. All the three sites were located in the front one third of HCV 5′ UTR. Further analysis on the secondary structure of this portion with RNA structure software revealed that the 3′ flanking sequence of the site C^67^-G^68^ formed a long single-strand region but the flanking sequence of C^20^-G^21^ or U^76^-G^77^ folded into a stable stem-loop structure (Figure 2), while the effect of long range annealing would make relevant regions less accessible for targeting [33,34]. Therefore, the region near C^67^-G^68^ may be more accessible for ribozyme binding.

Based on the above putative site (C^67^-G^68^), a custom guide sequence was designed, which was covalently linked to the 3′ termini of M1 RNA through an 88 nt-long bridge sequence, and a new targeting enzyme (i.e. M1GS-HCV/C_67_) for the 5′ UTR of HCV RNA was successfully constructed. In consideration of the influence of bridge sequence on the cleavage activities of M1GS as previously reported [35], a M1GS without a bridge, i.e. M1GS-HCV/C_67_*, was also constructed as a control. As shown in Figures 7 and 8, about 85% reduction of the expression level of HCV core protein and >1000-fold reduction of viral growth were observed in supernatant of cultured cells transfected with M1GS-HCV/C_67_ ribozyme. On the other hand, no obvious reduction of the levels of core gene expression and viral growth was observed in cells transfected with M1GS-HCV/C_67_*. Because M1GS-HCV/C_67_* contained an identical guide sequence with M1GS-HCV/C_67_ and thus had a similar binding affinity to target sequence. Therefore, the overall inhibition of viral gene expression and growth by M1GS-HCV/C_67_ was mainly due to the targeted cleavage by the ribozyme, as opposed to antisense or other nonspecific effects of the guide sequence.

It has been reported that cholesterol-modified siRNAs can be easily bound to human serum albumin, and thus cholesterol modification has the potential to improve *in vivo* pharmacokinetic properties of RNA therapeutics and broaden their tissue biodistribution [36]. Therefore, M1GS RNA was modified in this study with cholesterol on the 5′ terminus of M1 RNA within the ribozyme, and the cholesterol-conjugated M1GS ribozyme (i.e. Chol-M1GS-HCV/C_67_) did not lose its cleavage activity *in vitro* (Figure 5 Lane 4). Furthermore, similar to the unconjugated M1GS RNAs, Chol-M1GS-HCV/C_67_ was able not only to efficiently inhibit HCV gene expression in transiently transfected Huh7.5.1 cells (Figure 6 Lane 5 and Figure 7 Lane 4) but also to significantly reduce viral titers in the culture supernatant (Figure 8). Together, our data demonstrate the successful use of an M1GS ribozyme in the inhibition of HCV multiplication and provide an insight into the potential of M1GS-base therapeutics against HCV infection.

## Abbreviations

HCV: Hepatitis C virus; GS: Guide sequences; UTR: Un-translated region; MOI: Multiplicity of infection.

## Competing interests

The authors declare that they have no competing interests.

## Authors’ contributions

XLM designed the study, carried out the main experiments, analyzed results and drafted the manuscript. XFL initially conceived of the study. XJM analyzed the data and helped to edit the manuscript. ZWH and CCZ participated in gene cloning and sequence alignment. JGW helped for establishing the HCV culture system. WJZ and GL participated in the design of the study and the critical view of manuscript writing, they were the co-corresponding authors. All authors read and approved the final manuscript.
